# The Incidence of Other Gastroenterological Disease following Diagnosis of Irritable Bowel Syndrome in the UK: A Cohort Study

**DOI:** 10.1371/journal.pone.0106478

**Published:** 2014-09-19

**Authors:** Caroline Canavan, Timothy Card, Joe West

**Affiliations:** Division of Epidemiology and Public Health, University of Nottingham, Nottingham, United Kingdom; University Hospital Llandough, United Kingdom

## Abstract

**Background:**

Guidelines recommend Irritable Bowel Syndrome (IBS) diagnosis and management in primary care with minimal investigations; however little evidence exists regarding risk of organic gastrointestinal conditions following diagnosis of IBS and how such risks vary over the long term. This study assesses excess incidence of coeliac disease, inflammatory bowel disease (IBD) and colorectal cancer (CRC) and variation with age and time after IBS diagnosis.

**Methods:**

IBS patients and controls were identified within the UK Clinical Practice Research Dataset. Incidence rates were calculated and stratified by age and time since IBS diagnosis with incident rate ratios generated.

**Results:**

Fifteen years after IBS diagnosis there is a significant cumulative excess incidence of coeliac disease, IBD and CRC in IBS of 3.7% compared to 1.7% in controls. For every 10000 patient years, IBS patients experienced an additional 4 diagnoses of coeliac disease, 13 of IBD and 4 CRCs. In each condition peak excess incidence was in the 6 months following diagnosis. After one year, increased incidence of coeliac disease remained consistent without variation by age. IBD incidence fell slowly, with higher rates in those under 30. CRC incidence was increased only in patients aged 30 to 74 during the first 5 years.

**Conclusion:**

Some IBS patients later receive organic gastrointestinal diagnoses, with the early excess incidence likely detected during diagnostic investigation at the time of IBS diagnosis. More than 5 years after IBS diagnosis there is no increased risk of CRC compared to the general population, but a small excess risk of coeliac disease and IBD persists. Overall, though our findings provide reassurance that non-specialists, especially those in primary care, are unlikely to be missing an organic condition in the majority of their patients. This suggests that current guidelines suggesting avoidance of universal referral for these patients are appropriate.

## Introduction

Irritable bowel syndrome (IBS) is a chronic functional condition affecting about 11% of the global population [1]. It is clinically heterogeneous and patients present with various combinations of abdominal pain, altered bowel habit and bloating, but there is no biomarker or unifying structural abnormality to allow definitive diagnosis. Consequently diagnostic criteria have been developed to enable diagnosis based on symptom profile. Current international consensus criteria (Rome III) recommend diagnosing IBS without extensive investigations to exclude other conditions [2]. Alongside these recommendations, there is increasing emphasis on diagnosing and managing IBS within primary care [3], [4].

Despite this, 10–50% [5]–[7] of patients with IBS are referred to secondary care, with many gastroenterologists and generalists still considering IBS a diagnosis of exclusion [8]. Some referrals are due to concern about organic conditions that share similar symptomatology [7], [8], such as cholecystitis, pancreatic insufficiency, endometriosis, lactose intolerance, bile acid malabsorbtion and small bowel bacterial overgrowth. Three conditions of particular interest are coeliac disease, inflammatory bowel disease (IBD) and colorectal cancer (CRC). Currently little is known about the likelihood of a patient having one of these conditions if they have symptoms that suggest IBS [9]. Even less is known about the risk of patients subsequently being diagnosed with these conditions after being diagnosed with IBS. A small meta-analysis suggests prevalence of coeliac disease in patients diagnosed with IBS is around 4 times the population rate [10], but there are no large cohort studies considering this. Incidence rates of IBD and CRC in patients with IBS have previously been calculated, with estimates of IBD incidence between 8 [11] and 16 [12] times that in the general population and no increased incidence of CRC [12]. These studies, however, have short follow up periods and in one the study population were service personnel, unlikely to be a representative sample. Consequently, changes in risk of diagnoses of IBD, coeliac disease and CRC over a long time period after a diagnosis of IBS in a general population has not been studied and variations in risk by age and sex are unknown. A community based study followed 112 patients with IBS for a median of 29 years after diagnosis to assess incidence of organic gastrointestinal disease and found 3.5% were subsequently diagnosed with gastrointestinal cancer between 13 and 30 years later. They did not compare this to diagnoses in the general population to assess any excess risk and there was no incident IBD or coeliac disease [13].

We have therefore conducted a study within contemporary UK practice to determine the risk of diagnosis of coeliac disease, IBD and CRC over 15 years following IBS diagnosis and determined variation in risk according to time since diagnosis, age and sex. To aid better clinical decision making we have determined risk in absolute as well as relative terms.

## Methods

Data were taken from the Clinical Practice Research Datalink (CPRD). CPRD is an anonymised longitudinal dataset of over 13 million medical records from over 640 primary care practices across the UK, collected prospectively from routine care since 1987 [14], [15]. Almost two thirds of the practices are linked to the National Health Service Hospital Episode Statistics (HES), providing secondary care data from NHS hospitals in England since 1989 [15], [16]. Only practices with HES linked CPRD records and individuals with records audited to acceptable research quality [14] were included. This project was given ethical approval by the CPRD Independent Scientific Advisory Committee (protocol approval reference 12_047R) and all data was anonymised and de-identified by CPRD before release to us and prior to analysis. For this study we drew data from the July 2012 version of CPRD. All selections and definitions listed below were made using code lists available on request from the corresponding author.

### IBS population

People with IBS were identified by a Read code for IBS in their CPRD record for either a clinical or referral episode. Validity of these codes has been established by Huerta et al who contacted the primary care physicians of patients with Read codes consistent with incident IBS in CPRD and found records to be accurate in 77% of cases [17]. The purpose of the study is to examine the correlates of a GP diagnosis of IBS rather than those of the Rome criteria [2], so our inability to validate our code lists against these criteria is of limited importance. This study was only concerned with diagnoses of coeliac disease, IBD or CRC after an initial diagnosis of IBS, so any patients with these diagnoses before an IBS diagnosis were excluded, as were patients aged under 18 or over 75 years at diagnosis. We considered the earliest date associated with an IBS episode to be the date of diagnosis, and this was when the patient entered the study. IBS diagnoses were considered incident when this date was at least one year after the patient began contributing prospective data to CPRD (a previously validated strategy) [18]. 112854 cases of IBS met these criteria. Exit from the study was the earliest of date of death, date of transferring out of the practice or last date of data collection for the CPRD from that practice.

### Control population

Individuals were eligible to be controls if they had no recorded diagnosis of IBS and no prescription for peppermint oil or the spasmolytics Meberverine and Alverine (three medications prescribed for treatment of IBS and rarely for other conditions). As the controls, by definition, had no IBS diagnosis they had no diagnosis date to define entry to study and start of follow up. To allow for this and provide a date with which to match them to cases a pseudodiagnosis date was generated. Each potential control was allocated a pseudodiagnosis date randomly during the period they were alive and contributing data to CPRD, and controls were frequency matched [19] by sex, and age at diagnosis/pseudodiagnosis in 3 age bands (18–29, 30–49, and 50–75). The control population was generated five times the size of the case population to provide enough power to detect differences in incidence and conduct the stratified analyses [20]. Exit was defined in the same way as in cases.

### Defining other gastrointestinal diagnoses

All coeliac disease [21] and IBD [22] cases were defined as previously described as individuals who had at least one clinical episode with a Read code for coeliac disease or IBD in their CPRD record. CRC [23] cases were identified through either a clinical episode in the CPRD with a CRC Read term code or an episode in the linked HES records with an appropriate ICD-10 code. Incident diagnoses were defined as those where the first episode with an appropriate code occurred after the IBS diagnosis (or pseudodiagnosis) date.

### Statistical analysis

All patients with prevalent coeliac disease, IBD or CRC at date of IBS diagnosis (or pseudodiagnosis), or diagnosed on the same day were excluded from the analysis of incidence of that disease. Patients without an IBD clinical code with prescriptions for mesalazine, azathioprine, mecaptopurine and rectal steroids are likely to have these therapies for IBD so were also excluded from IBD incidence analysis. Incidence rates of coeliac disease, IBD and CRC were calculated by dividing the number of cases occurring by the person years at risk and are presented per 10000 person years with 95% confidence intervals (CI) and calculated over whole follow-up and after excluding the 1^st^ year. Nelson-Aalen cumulative hazard plots were generated for each condition separately. Poisson regression was used to estimate rate ratios and 95% CIs for the purposes of comparing the IBS and control cohorts. Likelihood ratio tests were used to check whether interactions exist between the incidence rates of each condition and age, calendar time, sex and smoking, which might suggest subgroups in which particular care should be taken to check for alternative diagnoses. A p value of less than 0.05 was taken to indicate evidence of an interaction. Rates were stratified by current age (in bands 18–29, 30–49, 50–74, 75 and older) and calendar time since diagnosis and compared using rate ratios. As there were few coeliac disease and IBD events in those aged over 75 years, this age group was combined with those aged 50 to 74 years for these analyses. Conversely, there were few CRC events in those aged 18 to 29 years so this group was combined with those aged 30 to 49 years. For the absolute rate differences, the same age groupings were used across the three diseases to allow direct comparison. The IBD group was also stratified into disease type (Crohn's disease, ulcerative colitis and unspecified) to assess any differences.

## Results

### Cohort demographics

**Figure 1:**
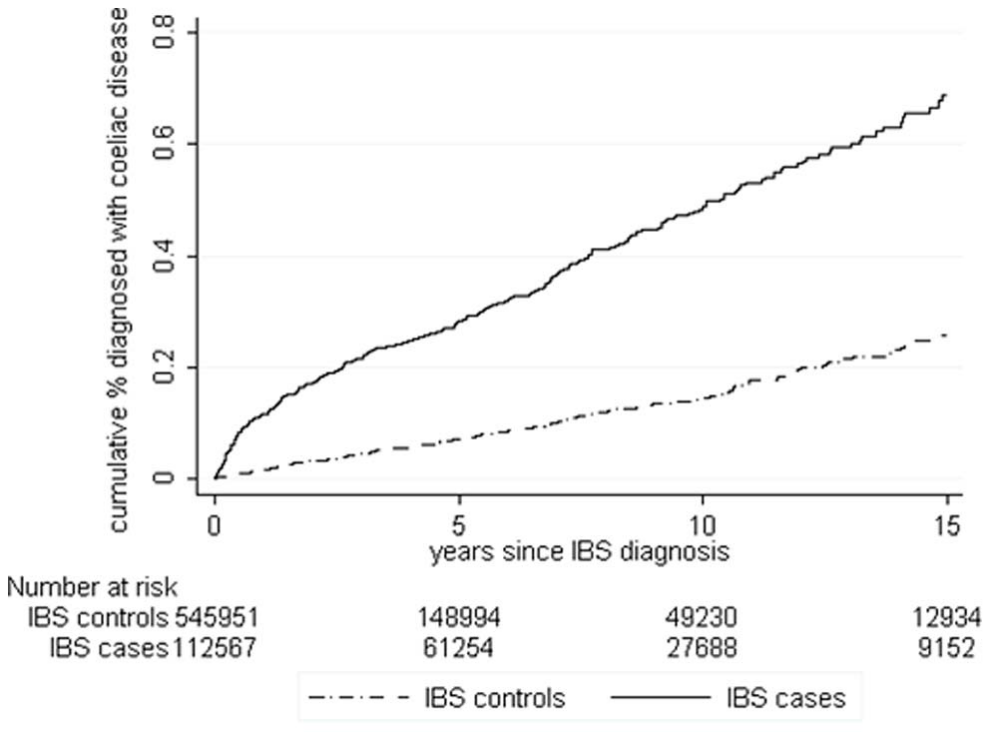
Cumulative frequency of coeliac disease in patients with IBS following IBS diagnosis compared to controls.

**Figure 2:**
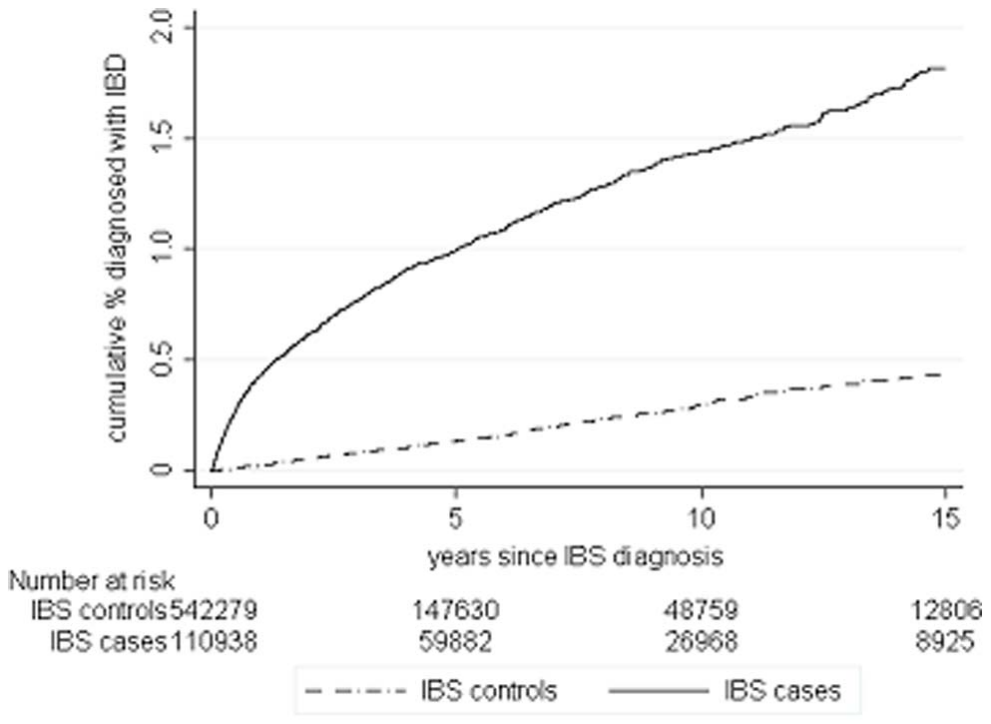
Cumulative frequency of IBD in patients with IBS following IBS diagnosis compared to controls.

**Figure 3:**
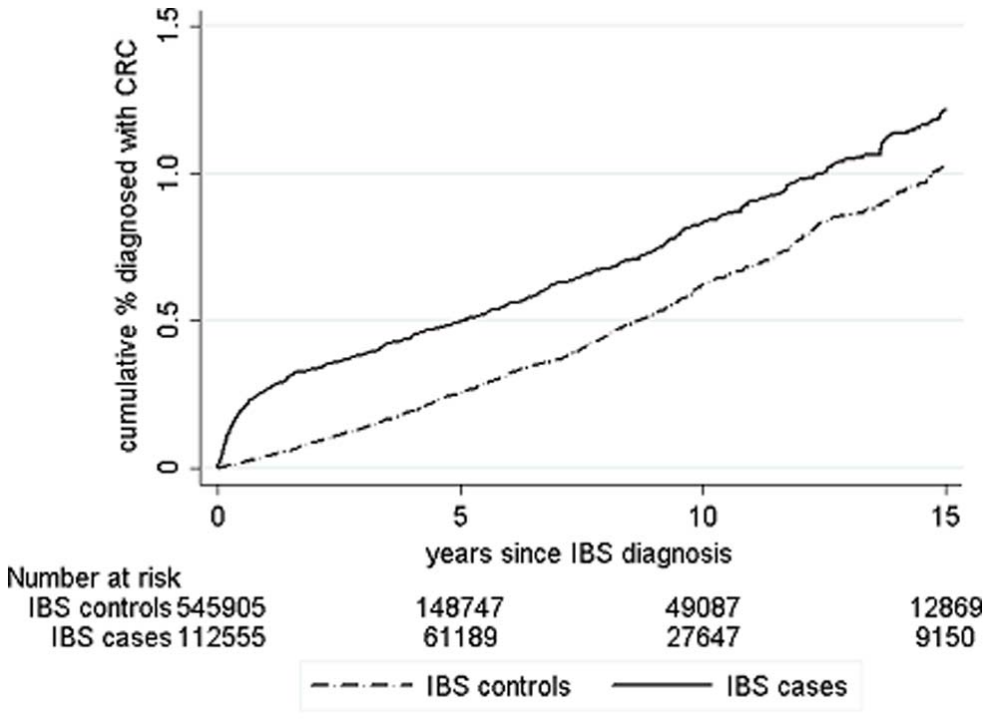
Cumulative frequency of coeliac disease CRC in patients with IBS following IBS diagnosis compared to controls.

There were 112854 cases of incident IBS aged between 18 and 75 years at diagnosis who contributed 733583 person-years of follow-up. Follow-up was more than five years for 61562 (55%), more than ten years for 27860 (25%) and 9224 cases (8%) had more than 15 years follow-up. To these, 546903 controls were frequency matched contributing 1990165 person-years of follow-up, with 149321 followed up for more than 5 years (27%) and 49368 for more than ten years (9%). The mean age at diagnosis of IBS (or pseudodiagnosis) was 42.9 years in the cases and 42.8 years in the controls (table 1). Figures 1, 2 and 3 shows the cumulative incidence of coeliac disease, IBD and CRC in patients following their IBS diagnosis over 15 years of follow-up compared to controls showing a steep increase in organic diagnoses in the first year or so after IBS diagnosis and then generally proportional risks. Combined, the overall cumulative incidence of being diagnosed with one of these conditions after IBS was 3.7%, compared to 1.7% in controls.

**Table 1:** Frequency and percentage of patients and controls stratified by age at IBS diagnosis and sex.

Age at IBS diagnosis	Total		IBS population		Control population	
	Cases (%)	Controls (%)	Male (%)	Female (%)	Male (%)	Female (%)
18–29	26954 *(23.88)*	126586 *(23.15)*	5883 *(21.83)*	21071 *(78.17)*	27398 *(21.64)*	99188 *(78.36)*
30–49	49121 *(43.53)*	246339 *(45.04)*	14141 *(28.79)*	34980 *(71.21)*	70635 *(28.67)*	175704 *(71.33)*
50–75	36779 *(32.59)*	173978 *(31.81)*	11478 *(31.21)*	25301 *(68.79)*	53322 *(30.65)*	120656 *(69.35)*
*Total*	112854	546903	31502 *(27.91)*	81352 *(72.09)*	151355 *(27.67)*	395.548 *(72.33)*

### Coeliac disease

During follow up 395 IBS cases and 299 controls were diagnosed with coeliac disease. Median time to coeliac disease diagnosis was 2.6 years following IBS diagnosis (IQR 0.7 to 6.8 years) and 3.5 years following pseudodiagnosis (IQR 1.31 to 7.07 years). Mean age at diagnosis of coeliac disease was 49 years in IBS patients and 53 years in controls.

Overall incidence of coeliac disease was 5.26 per 10000 person-years in IBS patients (95% CI 4.77 to 5.81) and 1.49 per 10000 person-years (95% CI 1.33 to 1.67) in controls, with a cumulative incidence of 0.7% of IBS patients after 15 years and 0.25% of controls (figure 1). The cumulative incidence of coeliac disease in IBS patients continues to diverge from that of controls even after 15 years (figure 1).

In IBS patients, an absolute rate difference of 4 extra coeliac disease diagnoses per 10000 person-years was seen over total follow-up compared to controls. The overall incident rate ratio (IRR) was 3.54 (95% CI 3.04 to 4.11), falling to 2.84 when the year after diagnosis was excluded (95% CI 3.38 to 3.38). Table 2 shows how the IRR varies with time since diagnosis and by the age of the patient. In the first 6 months after IBS diagnosis the incidence of coeliac disease was between 9.4 and 26.1 times higher than in controls, but this fell to between 2 and 7 times higher thereafter. This is reflected in the absolute rate difference (table 3) falling from between 12 to 17 additional coeliac disease diagnoses per 10000 person years in the first six months to a consistent excess after one year of between 3 and 4 cases per 10000 person years in those aged under 75 years. There was no significant increase in those aged over 75 years.

**Table 2:** Incidence rate of coeliac disease after IBS diagnosis and the incidence rate ratio.

Current age	Time since IBS diagnosis	IBS population						Control population						Rate ratio	95% CI
		*n* †	% ‡	N ††	Person time (years)	Incidence rate (per 10000 person years)	95% CI	*n* †	% ‡	N ††	Person time (years)	Incidence rate (per 10000 person years)	95% CI		
18–29	0–6 months	*20*	0.07	26976	12704	15.7	10.2–24.4	*3*	0.00	126742	49777	0.6	0.2–1.9	**26.1**	7.8–87.9
	6–12 months	*9*	0.04	23812	11128	8.1	4.2–15.5	*3*	0.00	80991	34616	0.9	0.3–2.7	**9.3**	2.5–34.5
	1–5 years	*23*	0.11	20707	49000	4.7	3.1–7.0	*7*	0.01	59342	104460	0.7	0.3–1.4	**7.0**	3.0–16.3
	5–10 years	*6*	0.10	6134	12943	4.6	2.1–10.3	*2*	0.02	9654	19058	1.0	0.3–4.2	4.4	0.9–21.9
	10 years to end of follow-up	*0*	0.00	467	400	0.0	-	*1*	0.14	736	629	15.9	2.2–110.0	-	-
30–49	0–6 months	*46*	0.09	50158	23983	19.1	14.4–25.6	*19*	0.01	251595	108719	1.7	1.1–2.7	**11.0**	6.4–18.7
	6–12 months	*15*	0.03	47953	22843	6.6	4.0–10.9	*17*	0.01	199764	90184	1.9	1.2–3.0	**3.5**	1.7–7.0
	1–5 years	*59*	0.12	50378	142459	4.1	3.2–5.3	*60*	0.03	178836	407334	1.5	1.1–1.9	**2.8**	2.0–4.0
	5–10 years	*39*	0.13	30147	92047	4.2	3.1–5.8	*29*	0.05	62419	163736	1.8	1.2–2.5	**2.4**	1.5–3.9
	10 years to end of follow-up	*9*	0.08	11224	36409	2.5	1.3–4.8	*6*	0.04	15233	42967	1.4	0.6–3.1	1.8	0.6–5.0
50 and older	0–6 months	*23*	0.06	37514	18078	12.7	8.5–19.1	*11*	0.01	177546	81018	1.4	0.8–2.5	**9.4**	4.6–19.2
	6–12 months	*11*	0.03	36673	17671	6.2	3.4–11.2	*13*	0.01	157476	73446	1.8	1.0–3.0	**3.5**	1.6–7.9
	1–5 years	*55*	0.13	41393	126085	4.4	3.3–5.7	*63*	0.04	161835	430279	1.5	1.1–1.9	**3.0**	2.1–4.3
	5–10 years	*44*	0.13	32915	111267	4.0	2.9–5.3	*38*	0.04	91991	277990	1.4	1.0–1.9	**2.9**	1.8–4.5
	10 years to end of follow-up	*36*	0.18	19968	73768	4.9	3.5–6.8	*27*	0.07	37207	125851	2.1	1.5–3.1	**2.3**	1.4–3.7

Incident rate ratios in bold are statistically significant and have a p-value <0.05.

† *n* is the number of people diagnosed with coeliac disease within the stratum;

‡ % is the proportion that these diagnoses represent within the stratum;

†† N is the total number of patients contributing time to the stratum. The rates are split by time since IBS was diagnosed and the current age of the patient. This means, for example, that the risk of coeliac disease for someone who is 34 and was diagnosed with IBS 6 years ago can be seen as 2.8 times greater than someone without IBS.

**Table 3:** Absolute incidence rate difference between IBS cases and controls for each disease stratified by current age and time since IBS diagnosis.

Current age	Time since IBS diagnosis	Coeliac disease		IBD		CRC	
		Absolute rate difference (per 10000 person years)	95% CI	Absolute rate difference (per 10000 person years)	95% CI	Absolute rate difference (per 10000 person years)	95% CI
18–29	0–6 months	**15.10**	4.83–16.45	**66.25**	51.66–80.84	**3.14**	0.06–6.23
	6–12 months	**7.20**	0.43–9.22	**38.26**	26.20–50.33	0.90	−0.86–2.66
	1–5 years	**4.00**	1.46–4.71	**18.51**	14.35–22.67	−0.57	−1.03– −0.12
	5–10 years	3.60	−0.87–6.51	**12.62**	5.26–19.99	-	-
	10 years to end of follow-up	-	-	9.62	−4.99–6.91	-	-
30–49	0–6 months	**17.40**	9.34–19.38	**55.21**	45.45–64.98	**17.44**	10.07–22.80
	6–12 months	**4.70**	1.66–7.98	**32.39**	24.61–40.16	**3.93**	1.18–6.67
	1–5 years	**2.60**	1.34–3.35	**11.73**	9.72–13.74	**0.94**	0.18–1.69
	5–10 years	**2.40**	0.13–2.60	**4.87**	2.83–6.91	0.05	−0.79–0.89
	10 years to end of follow-up	1.10	−0.51–3.05	**5.88**	2.49–9.27	−0.26	−1.96–1.44
50–74	0–6 months	**11.7**	6.4–16.9	**39.52**	29.88–49.16	**83.46**	69.09–97.84
	6–12 months	**4.7**	0.8–8.5	**16.93**	10.09–23.78	**24.65**	15.56–33.76
	1–5 years	**3.1**	1.8–4.4	**9.75**	7.61–11.90	**2.69**	0.50–4.89
	5–10 years	**2.7**	1.3–4.0	**6.49**	4.40–8.59	0.37	−4.85–2.59
	10 years to end of follow-up	**2.8**	0.8–4.8	**3.94**	1.57–6.31	−1.09	−4.14–1.95
75 and older	0–6 months	-	-	-	-	94.64	−939.23–496.49
	6–12 months	-	-	-	-	-	-
	1–5 years	−0.8	−3.9–2.3	6.14	−2.85–15.14	−4.59	−17.97–0.78
	5–10 years	2.0	−0.1–4.9	3.21	−1.79–8.21	4.58	−6.34–15.54
	10 years to end of follow-up	2.4	−1.5–6.3	**5.06**	0.05–10.10	−1.76	−12.01–8.49

Rates in **bold** are statistically significant with a p-value <0.05 The absolute rate difference indicates how many additional people would be expected to be diagnosed with each condition in a group with IBS, rather than the proportional difference that is presented in the rate ratio measure.

There was no statistically significant difference in the incidence of coeliac disease in men and women and no interaction between coeliac disease incidence and sex or smoking status.

### IBD

During follow-up 1184 IBS patients and 569 controls were diagnosed with IBD. The median time to diagnosis of IBD from IBS diagnosis was 1.7 years (IQR 0.49 years to 4.6 years) and 3.1 years (IQR 1.3 to 6.4 years) from pseudodiagnosis in controls. The mean age at IBD diagnosis was 45 years in IBS patients and 52 years in controls.

Overall incidence of IBD was 16.12 per 10000 person-years in IBS patients (95% CI 15.23 to 17.07) and 2.85 per 10000 person-years (95% CI 2.63 to 3.10) in controls. The cumulative incidence of IBD after 15 years was 1.9% of IBS patients and 0.5% of controls and the rates continue to diverge even after 15 years (figure 2).

Over the total follow-up there was an absolute rate difference of 13 extra cases of IBD per 10000 person-years in IBS patients compared to controls. The overall IRR was 5.63 (95% CI 5.11 to 6.24), which fell to 3.98 (95% CI 3.54 to 4.45) after the first year following IBS diagnosis was excluded. In the first 6 months after IBS diagnosis incidence of IBD in IBS was between 16.8 and 24.5 times that seen in the controls depending on age (table 4), an absolute rate difference of between 40 and 66 extra cases of IBD per 10000 person years (table 3). After 5 years, incidence of IBD in IBS patients remained between 2.6 to 5.0 times higher than in controls, with between 3 and 13 additional IBD diagnoses per 10000 person years depending on age.

**Table 4:** Incidence rate of IBD after IBS diagnosis and the incidence rate ratio.

Current age	Time since IBS diagnosis	IBS population						Control population						Rate ratio	95% CI
		*n* †	% ‡	N ††	Person time (years)	Incidence rate (per 10000 person years)	95% CI	*n* †	% ‡	N ††	Person time (years)	Incidence rate (per 10000 person years)	95% CI		
18–29	0–6 months	*87*	0.32	26789	12596	69.1	56.0–85.2	*14*	0.01	126414	49652	2.8	1.7–4.8	**24.5**	13.9–43.1
	6–12 months	*45*	0.17	26578	11010	40.9	30.5–54.7	*9*	0.01	80793	34527	2.6	1.4–5.0	**15.7**	7.7–32.1
	1–5 years	*100*	0.49	20472	48266	20.7	17.0–25.2	*23*	0.04	59180	104129	2.2	1.5–3.3	**9.4**	6.0–14.8
	5–10 years	*20*	0.33	6027	12669	15.8	10.2–24.5	*6*	0.06	9607	18946	3.2	1.4–7.1	**5.0**	2.0–12.4
	10 years to end of follow–up	*1*	0.22	458	389	25.7	3.6–182.6	*1*	0.14	729	621	16.1	2.3–114.3	1.6	0.1–25.5
30–49	0–6 months	*137*	0.28	49455	23624	58.0	49.1–68.6	*30*	0.01	250082	108046	2.8	1.9–4.0	**20.9**	14.1–31.0
	6–12 months	*78*	0.17	47191	22459	34.7	27.8–43.4	*21*	0.01	198503	89613	2.3	1.5–3.6	**14.8**	9.2–24.0
	1–5 years	*195*	0.39	49500	139731	14.0	12.1–16.1	*90*	0.05	177732	404694	2.2	1.8–2.7	**6.3**	4.9–8.1
	5–10 years	*72*	0.24	29513	89988	8.0	6.4–10.1	*51*	0.08	62018	162675	3.1	2.4–4.1	**2.6**	1.8–3.7
	10 years to end of follow–up	*30*	0.27	10945	35455	8.5	5.9–12.1	*11*	0.07	15130	42675	2.6	1.4–4.7	**3.3**	1.7–6.6
50 and older	0–6 months	*74*	0.20	37117	17689	41.8	33.3–52.5	*20*	0.01	177232	80144	2.5	1.6–3.9	**16.8**	10.2–27.5
	6–12 months	*34*	0.09	36285	17272	19.7	14.1–27.5	*22*	0.01	157305	72650	3.0	2.0–4.6	**6.5**	3.8-11.1
	1–5 years	*160*	0.37	43224	122965	13.0	11.1–15.2	*147*	0.09	168697	425404	3.5	2.9–4.1	**3.8**	3.0–4.7
	5–10 years	*101*	0.30	34179	108384	9.3	7.7–11.3	*89*	0.09	97062	274856	3.2	2.6–4.0	**2.9**	2.2–3.8
	10 years to end of follow-up	*50*	0.25	19916	71811	7.0	5.3–9.2	*35*	0.09	39420	124469	2.8	2.0–3.9	**2.5**	1.6–3.8

Incident rate ratios in bold are statistically significant and have a p-value <0.05.

† *n* is the number of people diagnosed with IBD within the stratum;

‡ % is the proportion that these diagnoses represent within the stratum;

†† N is the total number of patients contributing time to the stratum. The rates are split by time since IBS was diagnosed and the current age of the patient. This means, for example, that the risk of IBD for someone who is 34 and was diagnosed with IBS 6 years ago can be seen as 2.6 times greater than someone without IBS.

There was a significant interaction between incidence of IBD and current patient age (p <0.001). Whilst the risk ratios were not statistically significantly different according to age, because the underlying population incidence of IBD is higher in young people, the absolute rate difference in IBS compared to controls is significantly higher in those aged under 30 years after the first year following IBS diagnosis. There was a statistically significant interaction (p<0.001) between IBD incidence and sex. Overall incidence of IBD in men with IBS was 20.3 per 10000 patient years (95% CI 18.4 to 22.4) and 14.6 per 10000 patient years (95% CI 13.6 to 15.6) in women, but there was no statistically significant difference according to sex in controls. Stratification by IBD type showed no significant difference in any of the analyses. There was no interaction between the smoking status of IBS cases and controls and the incidence of IBD in each group.

### CRC

During follow up 708 people with IBS and 1148 controls were diagnosed with CRC. The median time from diagnosis of IBS to CRC diagnosis was 1.9 years (IQR 0.4 to 6.6 years), and 4.2 years in controls (IQR 1.8 to 7.9 years). Mean age at diagnosis was 63 years in IBS patients and 67 years in controls.

Overall incidence of CRC was 9.38 per 10000 person-years in IBS patients (95% CI 8.71 to 10.09) and 5.72 per 10000 person-years (95% CI 5.40 to 6.06) in controls. The cumulative incidence of CRC in IBS patients was 1.2% after 15 years and 1% in controls and rates began to converge soon after IBS diagnosis (figure 3).

Over the total follow-up there was an absolute rate difference of 4 extra CRCs per 10000 patient years in IBS patients compared to controls with an overall IRR of 1.64 (95% CI 1.49 to 1.80), which fell to 1.03 (95% CI 0.93 to 1.16) after the first year following IBS diagnosis was excluded, not statistically significantly different from the rate in the controls. In the first 6 months after IBS diagnosis the incidence of CRC was between 4 and 41 times higher than in controls depending on age, falling to between 1.3 and 1.7 times higher after one year (table 5). Patients who were aged under 30 only had an increased incidence of CRC in the first 6 months following IBS diagnosis. After this period and in those aged over 75 years at any time there was no significant increase in incidence of CRC compared to controls (table 3). For those aged 30 to 74, after 5 years there was no statistically significant difference in CRC incidence compared to controls, there is even a trend towards lower rates in patients with IBS.

**Table 5:** Incidence rate of CRC after IBS diagnosis and the incidence rate ratio.

Current age	Time since IBS diagnosis	IBS population						Control population						Rate ratio	95% CI
		*n* †	% ‡	N ††	Person time (years)	Incidence rate (per 10000 person years)	95% CI	*n* †	% ‡	N ††	Person time (years)	Incidence rate (per 10000 person years)	95% CI		
18–49	0–6 months	*47*	0.06	77254	36747	12.8	13.3–24.1	*5*	0.00	378738	158672	0.3	0.2–1.1	**40.6**	15.4–98.4
	6–12 months	*11*	0.02	71887	34035	3.2	2.4–8.1	*4*	0.00	281067	124949	0.3	0.2–1.2	**10.1**	3.1–31.4
	1–5 years	*26*	0.04	71235	191965	1.4	1.2–2.7	*42*	0.02	238462	512415	0.8	0.6–1.2	**1.7**	1.2–3.4
	5–10 years	*10*	0.03	36389	105387	0.9	0.6–2.0	*17*	0.02	72164	183078	0.9	0.6–1.7	1.0	0.5–2.3
	10 years until end of follow-up	*5*	0.04	11746	36993	1.4	0.6–3.3	*7*	0.04	16002	43691	1.6	0.8–3.4	0.8	0.3–2.6
50–74	0–6 months	*168*	0.45	37387	17891	93.9	80.7–110.0	*84*	0.05	177109	80459	10.4	8.4–12.9	**9.0**	6.9–11.7
	6–12 months	*60*	0.17	36053	17254	34.8	27.0–44.8	*73*	0.05	155574	72141	10.1	8.0–12.7	**3.4**	2.4–4.8
	1–5 years	*142*	0.35	40362	117682	12.1	10.2414.2	*380*	0.24	158585	405398	9.4	8.5–10.4	**1.3**	1.1–1.6
	5–10 years	*87*	0.29	30112	96815	9.0	7.3–11.1	*209*	0.25	84429	242501	8.6	7.5–9.8	1.0	0.8–1.3
	10 years to end of follow-up	*52*	0.32	16394	60034	8.7	6.6–11.4	*98*	0.31	31509	100508	9.8	8.0–11.9	0.9	0.6–1.2
75 and older	0–6 months	*1*	0.27	369	81	123.3	17.4–880.0	*1*	0.06	1551	349	28.7	4.0–200.0	4.3	0.3–68.8
	6–12 months	*0*	0.00	787	280	-	-	*3*	0.10	3011	1104	27.2	8.8–84.9	-	-
	1–5 years	*19*	0.53	3556	7503	25.3	16.2–39.7	*70*	0.61	11481	23397	29.9	23.7–37.4	0.8	0.5–1.4
	5–10 years	*44*	0.89	4935	13762	32.0	23.8–43.0	*94*	0.70	13344	34311	27.4	22.4–33.5	1.2	0.8–1.7
	10 years to end of follow-up	*31*	0.78	3971	13423	23.1	16.2–32.8	*61*	0.75	8110	24542	24.9	19.3–32.0	0.9	0.6–1.4

Incident rate ratios in **bold** are statistically significant and have a p-value <0.05.

† *n* is the number of people diagnosed with CRC within the stratum;

‡ % is the proportion that these diagnoses represent within the stratum;

†† N is the total number of patients contributing time to the stratum. The rates are split by time since IBS was diagnosed and the current age of the patient. This means, for example, that the risk of CRC for someone who is 34 and was diagnosed with IBS 6 years ago can be seen as being the same (1.0) someone without IBS.

There was a statistically significant interaction (p = 0.005) between CRC incidence and sex. Overall incidence of CRC in men with IBS was 14.9 per 10000 patient years (95% CI 13.3 to 16.6) and 7.3 per 10000 patient years (95% CI 6.7 to 8.1) in women. In controls, men still had higher rates but the difference was less (7.6 and 5.0 per 10000 patient years respectively). There was no interaction with smoking.

## Discussion

During the 15 years following a diagnosis of IBS, cumulative incidence of coeliac disease was 0.7%, 1.8% for IBD and 1.2% for CRC. This means the overall cumulative incidence of IBS patients receiving a diagnosis of at least one of these conditions was 3.7%, an absolute excess of 2.0% compared to people without IBS. In all three conditions, peak excess incidence was in the first 6 months following IBS diagnosis, followed by a marked drop after one year. Median time to each organic diagnosis was also less than three years following IBS diagnosis. This suggests that much of the excess incidence of organic gastrointestinal disease occurs during the diagnostic work-up, in these cases the IBS code does not reflect a final diagnosis but rather it is part of the clinical pathway to obtaining a final organic diagnosis. The most concerning differential diagnosis for physicians and patients is probably CRC [7] and our study has reassuringly shown that incidence is no higher than the general population in young or elderly patients, and for those aged 30 to 74 years the excess incidence is very low after one year and disappears after 5 years following IBS diagnosis. For coeliac disease and IBD the incidence remains higher in IBS patients than in controls at all ages, even after ten years with an IBS diagnosis, but the absolute excess risk although statistically significant, is small in absolute terms.

A major strength of our study is that it is the largest study of its type yet conducted, with power to consider not only the overall incidence rates, but to allow stratification by the individuals' sex, current age and time since IBS diagnosis. Such stratification allows us to more clearly define which of these organic gastroenterological conditions individual patients are at greater risk of. For example, a young male IBS patient is far more likely to have IBD than an elderly female one. A second strength is that the length of follow-up is greater than previous studies. Shorter studies are likely to be biased by the higher rates we have shown in the first 12 months, probably related to the diagnostic work-up for IBS. Our study adds 12 years of follow-up to the findings of Garcia-Rodriguez et al [12], allowing assessment of how incidence of organic disease changes over time since IBS diagnosis. Since most IBS diagnoses are made among those under 50 years such prolonged follow up is of particular importance. A further strength is that all subjects are taken from primary care. Since IBS is mainly diagnosed and managed within primary care, and recommendations suggest that this is optimal practice [2]–[4], our study design allows accurate assessment of the potential for missed organic gastrointestinal disease in patients treated in a typical manner rather than by those with a specialist interest in IBS. Many studies of IBS focus only on patients who attend secondary care who are likely to be systematically different to the majority of IBS patients who never consult secondary care. Studying all IBS patients identified within primary and secondary care removes this bias. Controls were defined as having neither a clinical episode with an IBS Read code attached nor a recorded prescription for peppermint oil, Mebeverine or Alverine. Although people with IBS take many medications, these are the only medications used almost exclusively in IBS. Excluding everyone who had used any medication potentially for IBS would more completely avoid misclassification of controls but would exclude everyone ever prescribed anti-depressants, anxiolytics, laxatives or anti-diarrhoeal agents, potentially selecting a control population with better health than the general population. Studies within the community suggest that around 50-70% of people with symptoms consistent with IBS never seek medical attention [24]. Thus some undiagnosed IBS will exist amongst controls but this should not invalidate our study since its aim is to address the incidence of organic gastrointestinal disease in patients recorded as having IBS within primary care.

Inaccurate coding presents a potential limitation. There is no definitive investigation finding to use as a gold standard for code list validation in IBS. For this study question, however, the lack of truly independent validation is less of a concern because the interest is in organic gastrointestinal diagnoses made in patients labelled as having IBS and consequently treated as such. Previous studies have shown coding of IBS within CPRD to be valid when compared to General Practitioner's opinion. Only 1% of coded incident IBS and 16% of prevalent IBS were not confirmed as IBS by the GP [25]. Similarly, 92% of IBD codes [22], over 90% of CRC codes [23] and a similar proportion of coeliac disease codes [26] have been shown to be valid. There is no reason to suppose that coding errors for events with CRC, IBD or coeliac codes are systematically different between IBS patients and controls, so this should not introduce any bias. These data do not allow assessment of how closely the Rome criteria were employed in establishing the IBS diagnosis or how closely the NICE clinical guidelines were followed. Differences in incidence of organic gastrointestinal disease according to diagnostic work up warrants further study. A further limitation of our study is that we have not considered the impact of confounding by socioeconomic status which might be associated with the risk of coeliac disease or IBD but is not significantly associated with IBS and so we think appreciable confounding unlikely [24], [27].

This is the first large primary care based cohort study to consider the incidence of coeliac disease in patients diagnosed with IBS. We found the cumulative incidence of coeliac disease was 0.7% of the incident IBS cases after 15 years, just under three times that in the controls. Our results are lower than the outcome of a systematic review of coeliac disease in IBS but show a similar increase compared to the general population. The review reported a pooled estimate of coeliac disease prevalence as 4% among 2278 IBS patients, four times that seen in controls [10]. One reason our cumulative incidence rate is lower may relate to as yet undiagnosed coeliac disease in our IBS cases. Also we have measured the cumulative incidence over time rather than prevalence. A study using an earlier version of CPRD in 2000 looked at the rate of diagnosis of IBD and CRC [12]. Then they had just under 3000 IBS cases and included only 5 years following IBS diagnosis. During this period they also saw initially high rates of IBD and CRC fall in the first year. They reported a crude incidence of 26.2 CRCs diagnosed per 10000 patient years [12], (considerably higher than we report over the complete follow-up now available, but similar to the incidence we found in the early follow-up period), and no overall excess compared to controls. They found an overall crude incidence of IBD of 17.8 cases per 10000 person years, 16 times the rate in controls [12] which is similar to our finding. Over their total follow-up they saw no reduction in the rate of IBD, however with increased follow-up we have shown that incidence does decrease over time, but still remains higher than in the general population. A US database study of IBS in over 9000 military personnel looked specifically at the risk of being diagnosed with IBD [11]. The average follow-up was 3.6 years and they found the rate of IBD diagnosis was 8 times higher than in the general population. The slightly lower excess of IBD in this study may reflect differences in medical practice between the USA and UK, but may also reflect a military population being relatively healthier than the population seen in routine UK primary care. Median time from IBS diagnosis to IBD diagnosis was 2.1 years, similar to our findings [11]. A low incidence (1.9%) of organic colonic disease was also found in a study reviewing colonoscopy outcomes in patients having the procedure to confirm IBS [28], with incidence of IBD and CRC not significantly increased compared to healthy controls [28].

The clinical implications here are that a clinical diagnosis of IBS in general practice is highly unlikely to lead to a serious gastrointestinal diagnosis in the following 15 years. Thus patients can be reassured, particularly after the relatively higher risk period immediately after IBS diagnosis has passed. This is especially true for CRC where a trend towards a reduced rate in IBS patients might be explained by IBS patients receiving a colonoscopy during their diagnostic work-up and any lesions being detected at that point. For coeliac disease and IBD, however, the incidence in IBS patients remains above that in the general population throughout. Previous analysis has suggested serological testing for coeliac disease would be cost-effective for all IBS patients at the time of diagnosis [29]. Our results will allow greater accuracy in estimating the parameters of the model underlying this analysis but they do not tell us the value of repeated testing for coeliac disease. As the absolute excess rate of coeliac disease is very small we cannot rule out the possibility that it reflects patients who are seronegative for coeliac disease when diagnosed with IBS seroconverting later on. Similarly for IBD, a recent report shows that over a quarter of patients with endoscopic remission of IBD complain of IBS-like symptoms [30], and this alongside findings that mesalazine may resolve symptoms in some IBS patients [31] could support a suggestion that symptoms compatible with IBS might in some instances reflect clinically undetectable IBD. Hence we cannot rule out the value of repeated investigation for this diagnosis either. Since the overall risk is very low, however, we believe any repeat investigation should be individually assessed.

In summary, our study shows that the vast majority of people who receive an organic diagnosis following a recording of IBS within primary care do so in the first year following their IBS diagnosis, which is clearly part of the diagnostic work up process. Five years after an IBS diagnosis, compared to people without IBS, there remains a small, but nonetheless important and statistically significant, excess risk of organic diagnoses. Overall our findings provide reassurance that non-specialists, especially in primary care who see most people diagnosed with IBS, are unlikely to be missing an organic condition in the majority of their patients. However younger IBS patients, particularly males, have a persistently increased risk of IBD, and patients of all ages still have a slight increased risk of having coeliac disease. This suggests that current guidelines suggesting avoidance of universal referral for these patients are appropriate.
